# Understanding health inequalities in Wales using the Blinder-Oaxaca decomposition method

**DOI:** 10.3389/fpubh.2022.1056885

**Published:** 2022-12-15

**Authors:** James Allen, Andrew Cotter-Roberts, Oliver Darlington, Mariana Dyakova, Rebecca Masters, Luke Munford

**Affiliations:** ^1^World Health Organization Collaborating Centre on Investment for Health and Well-Being, Public Health Wales, Cardiff, United Kingdom; ^2^School of Health Sciences, University of Manchester, Manchester, United Kingdom

**Keywords:** health inequality, health equity, health gap, mental well-being, life satisfaction, decompositions methods, contributing factors

## Abstract

**Background:**

Throughout Wales and the world, health inequality remains a problem that is interconnected with a wider and complex social, economic and environmental dynamic. Subsequently, action to tackle inequality in health needs to take place at a structural level, acknowledging the constraints affecting an individual's (or community's) capability and opportunity to enable change. While the ‘social determinants of health' is an established concept, fully understanding the composition of the health gap is dependent on capturing the relative contributions of a myriad of social, economic and environmental factors within a quantitative analysis.

**Method:**

The decomposition analysis sought to explain the differences in the prevalence of these outcomes in groups stratified by their ability to save at least £10 a month, whether they were in material deprivation, and the presence of a limiting long-standing illness, disability of infirmity. Responses to over 4,200 questions within the National Survey for Wales (*n* = 46,189; 2016–17 to 2019–20) were considered for analysis. Variables were included based on (1) their alignment to a World Health Organization (WHO) health equity framework (“Health Equity Status Report initiative”) and (2) their ability to allow for stratification of the survey sample into distinct groups where considerable gaps in health outcomes existed. A pooled Blinder-Oaxaca model was used to analyse inequalities in self-reported health (fair/poor health, low mental well-being and low life satisfaction) and were stratified by the variables relating to financial security, material deprivation and disability status.

**Results:**

The prevalence of fair/poor health was 75% higher in those who were financially insecure and 95% higher in those who are materially deprived. Decomposition of the outcome revealed that just under half of the health gap was “explained” i.e., 45.5% when stratifying by the respondent's ability to save and 46% when stratifying by material deprivation status. Further analysis of the explained component showed that “Social/Human Capital” and “Income Security/Social Protection” determinants accounted the most for disparities observed; it also showed that “Health Services” determinants accounted the least. These findings were consistent across the majority of scenarios modeled.

**Conclusion:**

The analysis not only quantified the significant health gaps that existed in the years leading up to the COVID-19 pandemic but it has also shown what determinants of health were most influential. Understanding the factors most closely associated with disparities in health is key in identifying policy levers to reduce health inequalities and improve the health and well-being across populations.

## Introduction

The effects of globalization have delivered a number of population health benefits, though not fairly distributed to all, with high unemployment levels, rising inequalities, and poor health outcomes remaining a problem (1). Health inequity is interconnected with wider and complex social, economic and environmental factors (2). Action to tackle inequalities and inequities in health outcomes must take place at a structural and system level, acknowledging the constraints affecting an individual's or community's capability and opportunity to enable change (3).

There are stark inequalities in health outcomes in the UK population and despite continuous research and recommendations to reduce these (4), issues remain in closing the health gaps. In Wales, differences in health outcomes have been observed for many years between the most and least deprived areas, and in some cases have worsened (5, 6). For example, the gap in death rates between the most and least deprived quintiles has slightly widened in recent years (7), largely driven by worsening life expectancy in the most deprived areas of Wales (8). The COVID-19 pandemic has also had an impact on health outcomes in Wales, particularly so among the most deprived (9).

Wales has a policy landscape which is well positioned towards helping the identification and tackling of health inequities. The Well-being of Future Generations (Wales) Act provides an overarching framework for understanding commitments toward reducing inequality (10). The Socio-economic Duty underlines the need to understand how public bodies' actions influence inequality and requires more equitable decision-making (11). “A Healthier Wales”, the Welsh Government's long-term plan for health and social care, outlines the need to measure health and well-being outcomes, and drives transformative change in places where these outcomes can be improved (12).

Despite the wealth of data exposing inequalities in health and their trends, and the policy commitment to reducing these, achieving a healthier and a more equal Wales, the specific drivers of the health gap remain poorly explored and understood. While the Social Determinants of Health (SDH) concept is well established (2), understanding the health gap composition and contributing factors is essential to identify its drivers and opportunities to reduce it.

The application of novel analytical methods to inform public health priorities are not only key to exploring the factors contributing to health inequities, but are crucial in identifying the policy levers to tackle them. With the much needed recovery from COVID-19 and the emergence of other social and economic challenges, such as the cost of living crisis, it is now more important than ever to understand the drivers of the health gap in Wales.

A promising method for exploring the health gap is the use of decomposition methods, which have the capacity to attribute sources of the compound construct of social inequality in health to independent conditions (13). While decomposition methods have been utilized to explore a range of issues in relation to health inequities – including age (14), gender (15), employment type (16) and socio-economic status (17) – they remain somewhat uncommon within public health (13). While some studies have analyzed self-reported or perceived health in the European context - specifically in Ireland (18), Latvia (19) and across Europe (20) – decomposition of the gaps in self-reported/perceived health remains an underutilized method. To the authors' knowledge, this is the first decomposition analysis using the Blinder-Oaxaca method of self-reported health in Wales and the UK.

This analysis is answering this need, aiming to explore and ‘decompose' the gaps in measures of self-reported health and well-being, and quantify their relationship with the wider determinants for healthy prosperous lives for all.

## Methods

This study is based on a secondary analysis of the National Survey for Wales (NSW) and was analyzed using Stata 14.

The NSW is conducted by the Office for National Statistics (ONS) on behalf of the Welsh Government. Initiated in 2012, the NSW covers a broad range of topics, and since 2016 has incorporated a number of other surveys including the Welsh Health Survey, the Arts in Wales Survey, the Welsh Outdoor Recreation Survey and the Active Adults Survey. Topics include:-

- Local area and environment
- Well-being and finances
- Housing
- Democracy and government
- Population health
- Internet and media
- Culture and Welsh language
- Sport and recreation
- Children and education
- NHS and social care

The NSW is conducted *via* a random sample (using the Royal Mail postcode address file) and a large scale telephone survey with a sample size of approximately 1,000 individuals per month. Prior to the COVID-19 pandemic (and for the time period of this study), the survey was conducted *via* face-to-face interviews.

Survey respondents are aged 16+ and not all questions in the survey are asked of the whole sample, e.g., some questions are only asked of pensioners/non-pensioner adults, this restricts what analysis is possible.

### Using a WHO health equity framework to decompose disparities in health

The WHO Regional Office for Europe's Health Equity Status Report identified five “essential conditions” (wider determinants) that impact health equity, namely, “health services”, “income security and social protection”, “living conditions”, “social and human capital” and “employment and working conditions” (1) (see Supplementary Figure 1 in the Supplementary material for the full definitions and examples).

These five conditions were used in conjunction with the Blinder-Oaxaca decomposition method to further understand the drivers of disparities in health.

The first step in being able to decompose the health gap according to the five essential conditions was to assemble a single dataset comprising all the questions and associated responses that were collected as part of the National Survey for Wales (NSW) between 2016–17 and 2019–20. From this combined dataset, we could then identify:

a) The questions most aligned to the five essential conditions
b) The questions allowing for stratification of the population into distinct groups where potentially significant gaps in health outcomes were present, and to generate the health outcomes themselves.

The combined survey data (2016–17 to 2019–20) yielded responses to over 4,200 questions by 46,189 people.

To be able to decompose the gap in health outcomes between distinct population groups according to the five essential conditions, questions from the surveys were categorized based on their ability to act as proxy variables for those five essential conditions.

We used a systematic approach to classifying the available questions; for each of the included questions, two reviewers independently attempted to categorize them according to the five essential conditions, health outcomes, and population stratification variables (or as irrelevant to the study). Where the two reviewers disagreed on the categorization of a variable, the final decision was made by consensus, with the final category of each question being mapped back onto the combined analysis dataset.

When determining which variables representing the five essential conditions, stratification factors and health outcomes should be included in the final decomposition analysis, a qualitative assessment must be made regarding both the perceived strength of a given question as a proxy for an essential condition, but also the corresponding sample size for the analysis resulting from its inclusion. As such, a balance must be struck between trying to include those variables that are felt to be the best indicators, while retaining enough observations to enable robust statistical analysis.

In order to determine this, a technical team made assessments of the questions and their alignment to each essential condition. This created a shortlist of the most appropriate questions for inclusion in the analysis under each condition. The final variable selection was then made by choosing the combination of variables from this shortlist that covered all five essential conditions, appropriate stratification factors and health outcomes, while minimizing the reduction in sample sizes due to the requirement for complete cases only.

In the case of stratification factors particularly, variables measured at an individual level were chosen in preference to area-based measures. For example, the ability of an individual respondent to save at least £10 a month was thought to be a better measure of relative financial deprivation than Welsh Index of Multiple Deprivation (WIMD), which will inherently capture less inter-person variation as it is based on the demographics of approximately 1,500 people living in an area. People living in more deprived areas are not necessarily deprived, however, we can say that someone who is unable to save at least £10 a month is likely less financially secure than someone who can.

### Data considerations

It was not possible to use questions that were not asked concurrently over the 4 year period as the decomposition analysis requires complete cases only.

An important consideration was which questions could be included in the analysis alongside one another. For example, if one question was only asked in the 2016–17 survey, and conversely, another question was only asked in the 2019–20 survey, these two questions could never be jointly included in the analysis, as a single respondent would have been unable to provide answers to both questions due to the cross-sectional design of the survey.

The wording of the questions must remain the same over the 4 year period to be included in the analysis. Although there were cases where questions in different years may have been in effect the same, or attempted to investigate the same issue, it was not possible to quantify how similar two questions were, or subsequently decide a threshold to determine if questions were similar enough to aggregate across survey years.

### Variables included in the decomposition

The final decomposition analysis included 16 variables aligned to the five essential conditions, which are categorized as follows:

- Employment and working conditions: excessive hours worked and job satisfaction
- Health services: satisfaction with health services
- Income security and social protection: not in paid work, use of food banks and trouble keeping up with bills
- Living conditions: satisfaction with local area, internet access, if the respondent was living in a single person household and if the respondent felt safe in the local area
- Social and human capital: highest qualification, sense of trust in their community, sense of community more broadly, if they volunteered and participation in sports and other activities

The outcomes considered for the decomposition were: reported poor or fair health, low mental well-being, and low life satisfaction (see Supplementary Table 1 for coding of variables).

The decomposition analysis sought to explain the differences in the prevalence of these outcomes in groups stratified by their ability to save at least £10 a month, whether they were in material deprivation, and the presence of a limiting long-standing illness, disability of infirmity. A full description of the survey questions included in the analysis and how they were mapped to analysis variables is shown in Supplementary Table 1. Any responses where the respondent either refused to answer the question, or did not know the answer to the question were omitted from the analysis.

Mapping of analysis variables was done to either demonstrate negative outcomes/ active participation in an activity, as a result of this, the polarity of coding is inconsistent across variables. This should be borne in mind when interpreting results. It should also be stated that if the reference/base category for predictor variables was changed, it could impact/change the results of the decomposition analysis.

### Statistical analysis

The survey design and subsequent data management means that the analyzed dataset considers the 4 years' worth of survey data as one time period, meaning a cross-sectional study design,

The logit models used a pooled form of the Blinder-Oaxaca decomposition that generates a two-fold decomposition which uses the coefficients from a pooled model over both groups as the reference coefficients, aligned with the work of Neumark (21) and Oaxaca and Ransom (22). This is partly due to an index problem, where it is not clear which regression co-efficient should be used as the reference.

The formula for the decomposition is as follows:


(1) $$\begin{array}{l} y^{Group\;1}-\;y^{Group\;2}=\text{Δ}x\beta^{P}+[x^{Group\;1}\left(\beta^{Group\;1}-\beta^{P}\right)\\ \;+x^{Group\;2}\left(\beta^{P}-\;\beta^{Group\;2}\right)]\; \end{array}$$

*y* = health variable of interest

*β* = coefficients

*x* = vector of underlying conditions

*β*^*P*^ = pooled coefficients

Predicted means for the outcome of interest is presented in percentage form, alongside a breakdown of the difference in predicted mean by explained/unexplained component. The explained component has been broken down further using the WHO health equity framework and presented in graphical form. Full breakdowns are presented in Supplementary Tables. Percentages shows how much of the total difference in a health outcome is accounted for by the level of observed covariates in the model. This follows a similar approach taken by Rahimi and Nazari (23).

Whether the prevalence of an outcome was significantly different from another was determined by whether the 95% confidence intervals were overlapping or not; statistical significance of the explained/unexplained component was determined by a significance threshold of α = 0.05.

## Results

### The health gap between those who are able to make a saving of at least £10/month and those who are not

**Figure 1:**
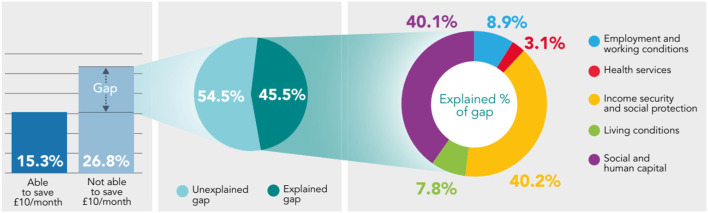
Decomposing the gap in prevalence of fair/poor health between those who are able to make a saving of at least £10/month, and those who are not using the Blinder-Oaxaca methodology, non-pensioner adults (aged 16–65), Wales, 2016–17 to 2019–20.

**Figure 2:**
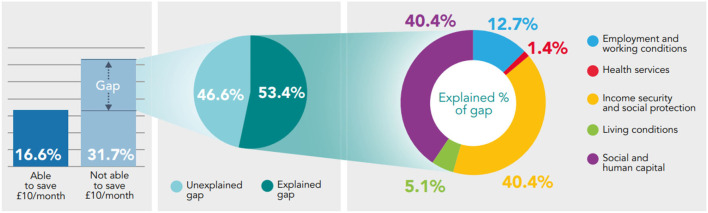
Decomposing the gap in prevalence of low mental well-being between those who are able to make a saving of at least £10/month, and those who are not using the Blinder-Oaxaca methodology, non-pensioner adults (aged 16–65), Wales, 2016–17 to 2019–20.

**Figure 3:**
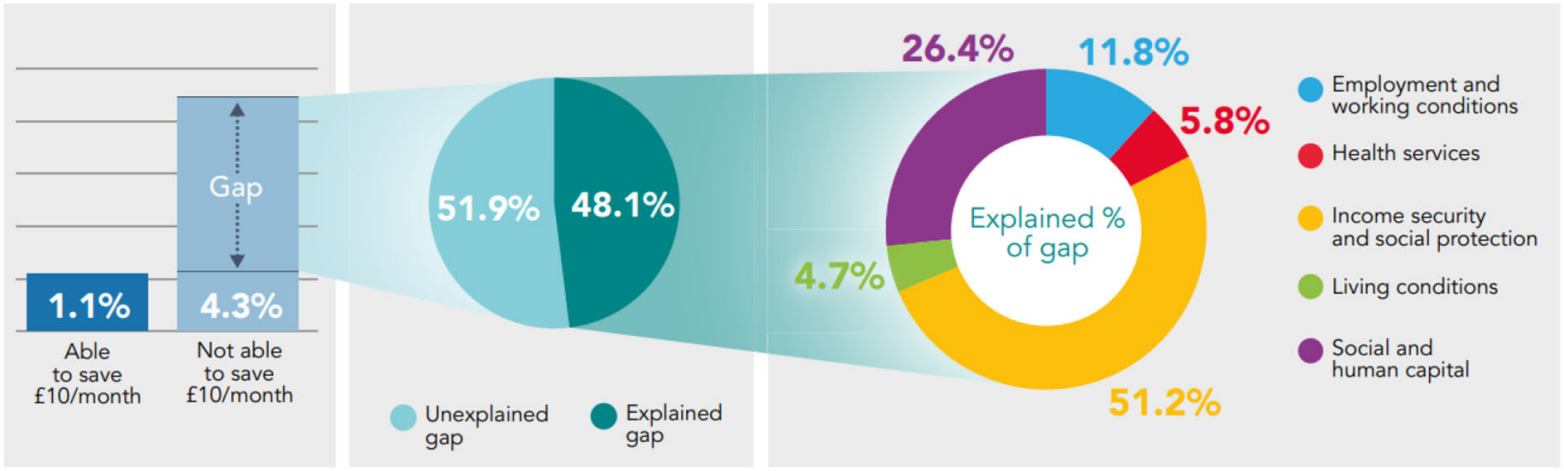
Decomposing the gap in prevalence of low life satisfaction between those who are able to make a saving of at least £10/month, and those who are not using the Binder-Oaxaca methodology, non-pensioner adults (aged 16–65), Wales, 2016–17 to 2019–20.

This analysis quantifies the prevalence of low mental well-being, low life satisfaction and fair/poor health in those who are able to make financial savings and those who are not (Figures 1–3). 31.7% of survey respondents who were not able to make savings reported low mental well-being, compared to 16.6% of those who were able to save, a significant difference of 15.1 percentage points. 4.3% of survey respondents not able to make savings reported low life satisfaction compared to 1.1% of respondents who were able to make savings, a significant difference of 3.2 percentage points. 26.8% of survey respondents who were not able to make savings reported being in fair/poor health, compared to 15.3% of respondents who were able to make savings, a significant difference of 11.5 percentage points.

Decomposing the gap in prevalence of self-reported health between those able to make a saving of at least £10/month and those who are not, reveals that Social and Human Capital (26.4–40.4% of explained component) and Income Security and Social Protection (40.2–51.2% of explained component) are the essential conditions accounting the most for the differences in fair/poor health, low mental well-being and low life satisfaction. The Living Conditions and Health Services are the essential conditions accounting the least for differences in health (Figures 1–3).

From the gap in prevalence of fair/poor health between those who are able to save at least £10/ month and those who are not (11.5 percentage point difference) – 45.5% can be explained by systematic differences in the essential conditions; and 54.5% remains unexplained, both statistically significant (*p* < 0.05). From the explained component, Income Security and Social Protection and Social and Human Capital accounts the most, 40.2% and 40.1% (both statistically significant *p* < 0.05), respectively; while Living Conditions (7.8%; *p* = 0.05) and Health Services (3.1%; *p* = 0.08) accounts the least (Figure 1).

From the gap in the prevalence of low mental well-being (15.1 percentage point difference) – 53.4% can be explained by systematic differences in the essential conditions; and 46.6% remains unexplained (both components statistically significant (*p* < 0.05). From the explained component, Social and Human Capital (40.4%; *p* < 0.05) and Income Security and Social Protection (40.4%; *p* < 0.05) account the most for differences in health and have equal shares, relative to the other essential conditions. Health Services accounts the least for differences in fair/poor health (1.4%; *p* = 0.24) (Figure 2).

While the gap in prevalence of low life satisfaction (Figure 3) is smaller than the gap observed in low mental well-being and fair/poor health (Figures 1, 2), the prevalence of low life satisfaction is still approximately four times higher in those that are not able to make a saving than those who are, and still significantly higher. From this, 48.1% can be explained (*p* < 0.05) by systematic differences in the essential conditions; while more than half of the gap (51.9%) remains unexplained (*p* = 0.08). Of the explained component, Income Security and Social Protection (51.2%; *p* < 0.05) and Social and Human Capital (26.4%; *p* < 0.05) accounts the most for the differences in low life satisfaction; while Living Conditions accounts the least, 4.7% (*p* = 0.26) (Figure 3).

### The health gap between those who report being in material deprivation and those who do not

Breaking down self-reported measures of health by whether the respondent is in material deprivation or not reveals stark health gaps. The prevalence of negative health outcomes (fair/poor health, low mental well-being and low life satisfaction) is at least twice as high in those who report being in material deprivation compared to those who do not: 22.9 significant percentage point difference in the prevalence of low mental well-being; 14.7 significant percentage point difference in fair/poor health; and a 5.2 significant percentage point difference in low life satisfaction.

**Figure 4:**
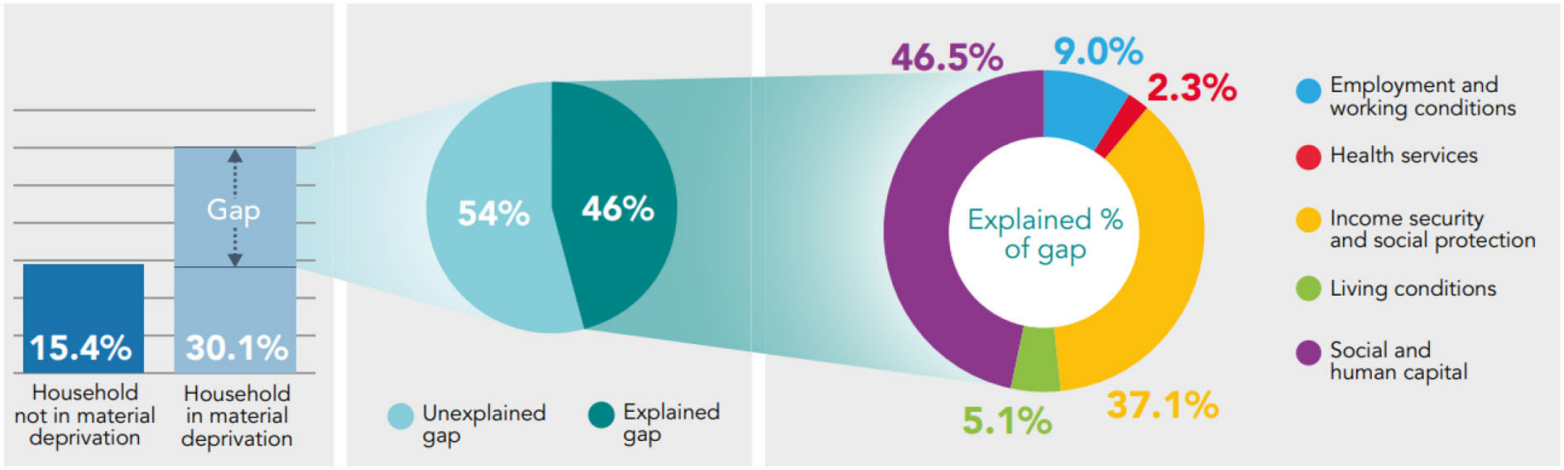
Decomposing the gap in prevalence of fair/poor health between those who report being in material deprivation and those who do not using the Binder-Oaxaca methodology, persons aged 16+, Wales, 2016–17 to 2019–20.

**Figure 5:**
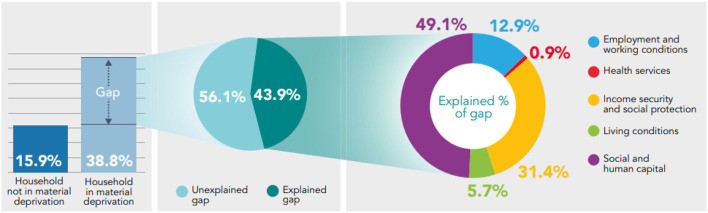
Decomposing the gap in prevalence of low mental well-being between those who report being in material deprivation and those who do not using the Binder-Oaxaca methodology, persons aged 16+, Wales, 2016–17 to 2019–20.

**Figure 6:**
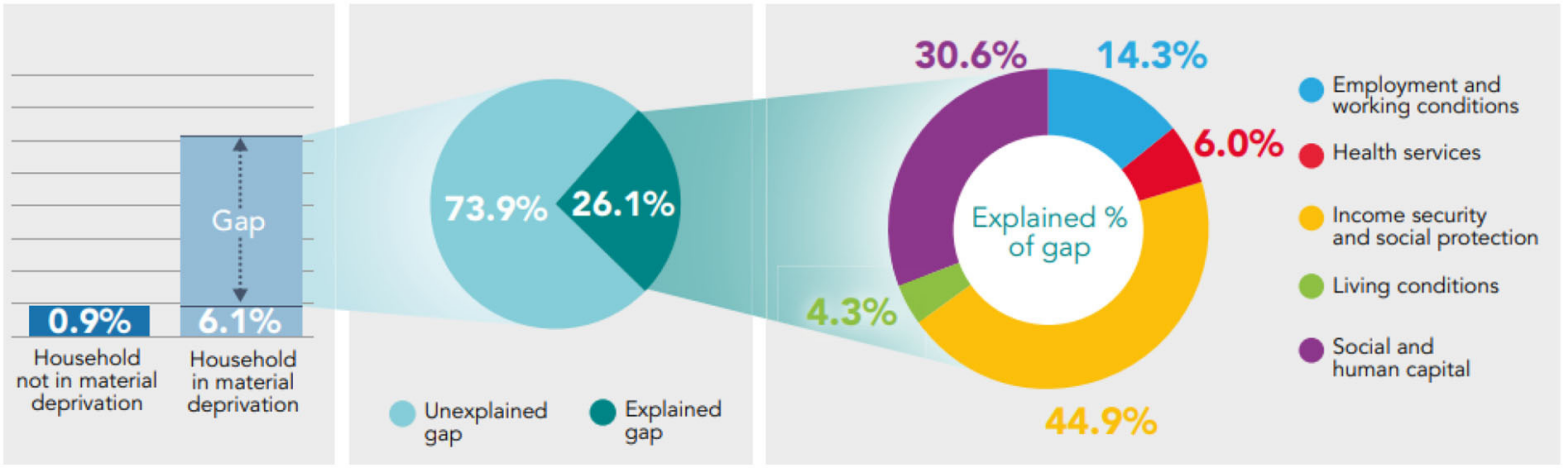
Decomposing the gap in prevalence of low life satisfaction between those who report being in material deprivation and those who do not using the Binder-Oaxaca methodology, persons aged 16+, Wales, 2016–17 to 2019–20.

The decomposition analysis of the health gaps observed between those in material deprivation and those who aren't shows that most of the gap cannot be explained by the model (54–73.9%). Of the proportion that can be explained, Social and Human Capital and Income Security and Social Protection accounts the most for differences in health. In the majority of scenarios, Health Services accounts the least for the differences in health (Figures 4–6).

46% (*p* < 0.05) of the gap in fair/poor health between those who report being in material deprivation and those who do not can be explained by the model; 54% (*p* < 0.05) of the gap remains unexplained. From the explained component, Social and Human Capital and Income Security and Social Protection accounts the most for differences in fair/poor health, 46.5 and 37.1%, respectively, both statistically significant (*p* < 0.05) (Figure 4).

Social and Human Capital accounts the most for differences in low mental well-being (49.1% of explained component; *p* < 0.05); Health Services accounts the least (0.9% of explained component; *p* = 0.36) (Figure 5). Income Security and Social Protection accounts the most (~44.9% of explained component; *p* < 0.05) for differences in low life satisfaction and Living Conditions accounts the least (4.3% of explained component; *p* = 0.5) (Figure 6).

### The health gap between those who report a limiting long-standing illness, disability or infirmity and those who do not

**Figure 7:**
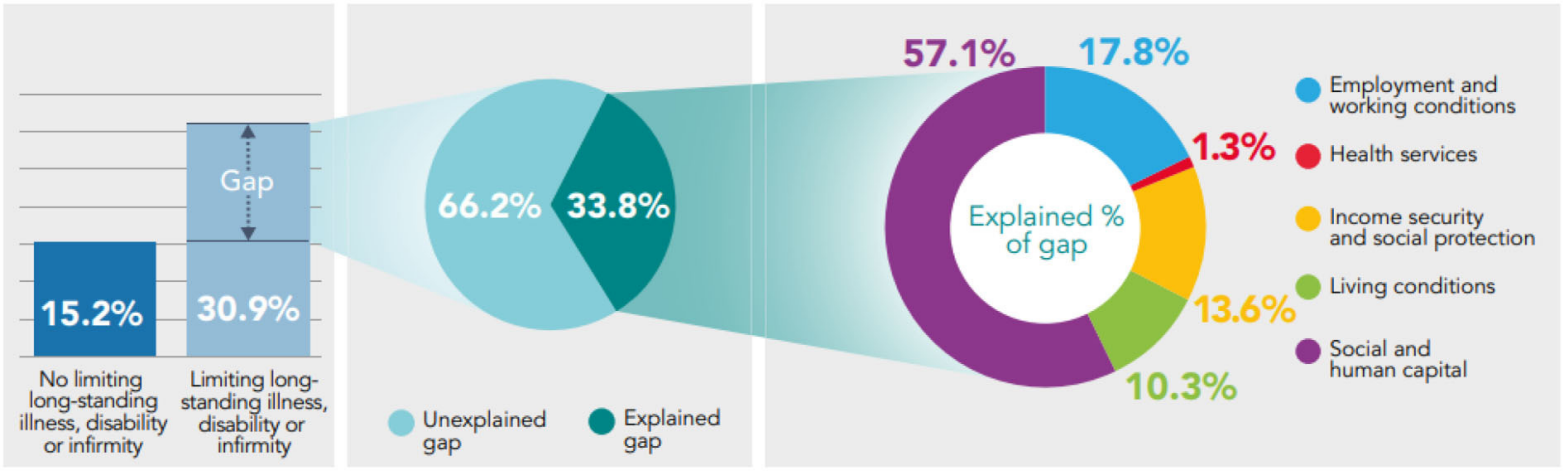
Decomposing the gap in prevalence of low mental well-being between those reporting a limiting long-standing illness, disability or infirmity, and those who do not using the Binder-Oaxaca methodology, persons aged 16+, Wales, 2016–17 to 2019–20.

**Figure 8:**
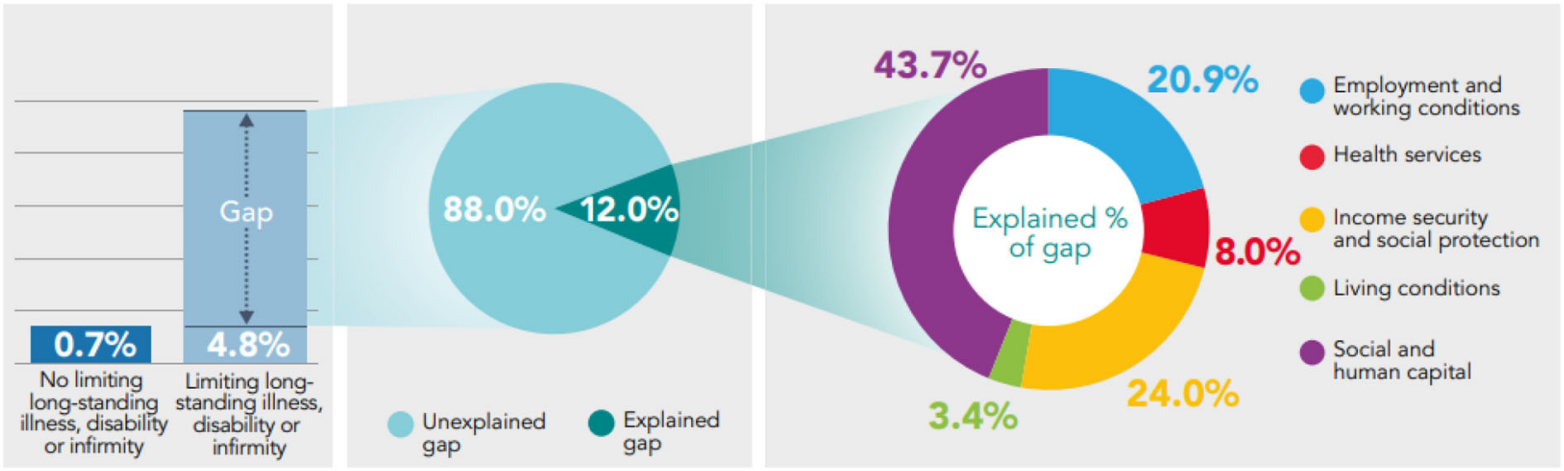
Decomposing the gap in prevalence of low life satisfaction between those reporting a limiting long-standing illness, disability or infirmity, and those who do not using the Binder-Oaxaca methodology, persons aged 16+, Wales, 2016–17 to 2019–20.

Analysis shows that the prevalence of low mental well-being and low life satisfaction is significantly higher in those who report a limiting long-standing illness, disability or infirmity, compared to those who do not (Figures 7, 8). 4.8% of survey respondents who report a limiting long-standing illness, disability or infirmity report low life satisfaction, compared to 0.7% in respondents who do not, a significant difference of 4.1 percentage points. The prevalence of low mental well-being is also higher in those reporting a limiting long-standing illness, disability or infirmity (30.9%) compared to those not reporting (15.2%), a significant difference of 15.7 percentage points.

Exploring the health gap experienced between those who report a limiting long-standing illness, disability or infirmity and those who do not, shows that a large proportion of the gap in low mental well-being and low life satisfaction cannot be explained by the model: 66.2% of the gap observed in low mental well-being and 88% of the gap in low life satisfaction is unexplained by the model (*p* < 0.05). Of the explained component, Social and Human Capital accounts the most for differences in health (*p* < 0.05) (Figures 7, 8).

Decomposing the gap in low mental well-being shows that from the explained component, Social and Human Capital accounts the most for the difference in low mental well-being (57.1%; *p* < 0.05), and Health Services accounts for the least, 1.3% (*p* = 0.3) (Figure 7).

Of the small proportion of the health gap in low life satisfaction that can be explained (12%), Social and Human Capital and Income Security and Social Protection accounts the most for differences in health, 43.7% (*p* < 0.05) and 24% (*p* < 0.05), respectively. Health Services and Living Conditions accounts the least for differences in low life satisfaction, 8% (*p* = 0.103) and 3.4% (*p* = 0.71), respectively (Figure 8).

## Discussion

Exploring the persisting inequalities in Wales has revealed stark differences in health outcomes before COVID-19. Since the start of the pandemic there is growing evidence on the unequal impact COVID-19 has had on different population groups (9).

The persistent gaps and fragmentation in public health data and the need to invest in strong health information systems has been acknowledged by the WHO (24). Wales (and the wider UK) benefits from collecting robust data on demographics, health outcomes and lots of the wider determinants (essential conditions) needed for health. This provides a data landscape comparatively richer than other countries in the WHO European Region.

The decomposition analysis has revealed what more can be done in the population health intelligence field to gain a deeper understanding to what is driving health inequity in Wales and beyond. It has quantified the health gaps that exist in Wales between population groups, whether the groups are defined financially (by their ability to make financial savings); materially (by whether they are materially deprived); or physically (by whether they have long-standing limiting illness, disability or infirmity). Reporting of negative health outcomes (fair/poor health, low mental well-being, and low life satisfaction) is found to be significantly higher in those who are disadvantaged.

The applied decomposition analysis has not only quantified the health gap, but it has also generated a unique insight into the drivers (essential conditions) that contribute the most to the differences in health within defined population groups. This can allow policy and decision-makers to see the potential of applying this methodology further to identify policy areas most likely to influence the health gaps and reduce inequities in health.

The analysis uses the NSW which surveys Welsh residents aged 16+. This means that the health gaps measured are only representative of the Welsh adult population and do not capture how the wider determinants of health are associated with health outcomes in children aged < 16.

The study uses self-reported data meaning survey respondents may not provide answers that are accurate and may be more likely to give responses that are socially desirable. For some variables, recognized scales are used as a measurement tool e.g. well-being is measured using the Warwick-Edinburgh Mental Well-being Scale (WEMWBS). For other variables, Likert scales have been used, where the respondent is provided with five possible answers to a statement indicating positive-to-negative strength. However, problems with using self-reported health as a method of measurement remain, particularly in that its relative nature makes it susceptible to changing contexts. For example, a recent analysis from the Netherlands on self-reported health pre and post COVID-19 pandemic found improvements in the levels of reported health. However, without substantial improvements in the health services or general living conditions, these are theorized to be due to uninfected individuals evaluating their health more positively than under normal circumstances – partly through social comparison with infected individuals (25).

The analysis uses survey data ranging from 2016–17 to 2019–20, which means that any health gaps quantified have not taken into account the impact of the COVID-19 pandemic in exacerbating inequalities (26), which would likely impact the results of the analysis.

Variables measured using the NSW have been used as proxies for each of the essential conditions, which, taken in isolation are difficult to measure (e.g., there are limited variables within the NSW that align to Health Services). Mapping the variables to the essential conditions is detailed in the methods section and need to be considered when interpreting results; full details of coded variables and analytical values used are presented in Supplementary Table 1.

It should be noted that although we used a systematic approach to categorize the available questions into those aligning to the five essential conditions to minimize bias, ultimately the decision is a subjective one based on our judgement, and the included variables, and associated results, should be considered in that context.

Data management and survey design means that the analyzed dataset considers the 4 years' worth of survey data as one time period, meaning a cross-sectional study design. This type of survey design limits the analysis to exploring strengths of associations between variables and no causality can be determined from the analysis.

The analysis reveals that there is variation in the extent to which “essential conditions” were represented in the survey. For example, there is significantly less variables aligning to Health Services compared to alignment to Social and Human Capital. This impacted our approach to analysis.

The data requirements of the methodology demand a large survey sample sizes and, in our experience, the inconsistency of survey questions over different years of survey data proves challenging. This has restricted what has been feasible in this exploratory analysis and it has limited the proxy variables chosen for each of the essential conditions.

In all scenarios, Social and Human Capital and Income Security and Social Protection account for the largest portions of differences in self-reported general health, mental well-being and life satisfaction. Health Services and Living Conditions are found to have a much smaller contribution. Our findings therefore align with long established linkages between income and health and well-being (27–30), and also with continued contemporary trends in self-reported health across Europe surrounding income (18, 19) and in North America surrounding education (16, 31). In addition, our findings support empirically derived evidence indicating the significant impact education (32) and income (17, 33) have on general health. However, the work of Sinha et al. (34) indicates that there are potentially better indicators of poor health than income, and that tackling persistent deprivation in dimensions such as housing conditions and social isolation may be more fruitful.

While income has been highlighted as a key driver in health gaps and health inequity in some studies in Europe – including in Ireland (18) and Latvia (19) – the time period focus of these studies was in the wake of the Great Recession, and related more to the effects of economic downturn on health. In addition, an analysis of self-reported health across Europe (20) indicated that decreasing household income had little effect on self-reported health, while decreasing employment and transitions to economic inactivity had a significant impact. Our analysis focuses on a slightly different contextual outlook, particularly economically tumultuous time periods [2016–17 in the wake of the European Union (EU) referendum; and 2019–20 with the looming fallout of the COVID-19 pandemic] that were not *yet* characterized by economic downturn. Our analysis also offers a unique lens in terms of context, highlighting the driving factors in health gaps in self-reported health in a somewhat fragile, post-industrial economy in Western Europe.

This analysis has shown that the health services alone cannot address the health gap in Wales and other sectors play a greater role in tackling them. These findings are consistent with the wider evidence base showing that it is the wider determinants (referred to as essential conditions throughout this paper) that exert the greatest impact on health and well-being. Studies have shown that only 20% of a person's health outcomes are attributed to access to good quality health care and have highlighted the crucial role of communities and local settings (35, 36).

It is also important to note that the health sector is delivering more than clinical services; it also delivers public health (prevention) services, as well as its strong links with the wider economic, social and environmental domains, such as education and employment, all of which are interconnected with population well-being and health equity.

The National Health Service (NHS), often referred to as an “anchor”, can go beyond direct healthcare and look to influence the wider determinants of health by purchasing locally for social benefit, use buildings and spaces to support communities, widen access to quality work, work more closely with partners and reduce its environmental impact (37, 38).

### Application for investment prioritization

Comparing government or local expenditure to the drivers of health inequity, can provide useful insights into where further resources and investment can be shifted and targeted to make the most difference.

Within the total identifiable expenditure in Wales (2019/20), the largest category is Social Protection which, when broken down further, covers expenditure on personal social services, unemployment benefits etc., accounting for 43% (£14.8 billion) of total expenditure in Wales. It is followed by the Health sector which accounts for 23% (£8.3 billion) of total expenditure.

**Figure 9:**
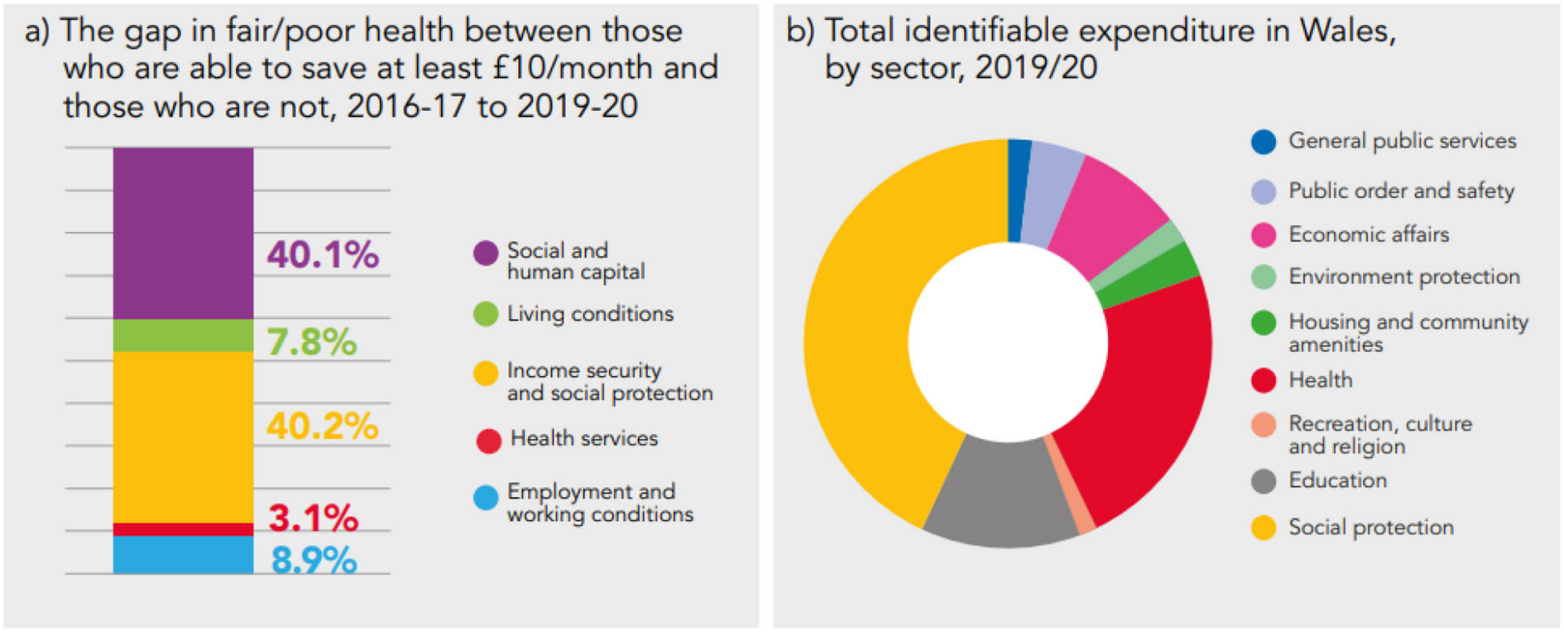
Comparing findings from the decomposition of the health gap **(a)** to government expenditure data **(b)**.

Triangulation of the results from the decomposition analysis and Wales' expenditure data has the potential to reveal alignment or mismatch; and can provide a useful lever for informing and strengthening the case for investing in well-being and health equity (Figure 9). This analysis suggests that with a longer term view, health gaps can be tackled through greater investment in prevention and the wider determinants of health, rather than reactive investment in the provision of clinical (care) services (39).

This study has outlined the challenges and opportunities in applying the decomposition analysis method.

Further exploration, research, data gathering and analysis is needed, engaging with and involving relevant groups and communities, to understand the health gap and its drivers, for example:

- Exploring the application of the decomposition methodology to linked data to allow for stronger alignment between the WHO HESRi framework and individual-level variables, particularly those that represent health services;
- Exploring other stratification factors, for example, those that capture deprivation, but are measured on an individual-level;
- Applying the methodology to longitudinal survey data (e.g., Understanding Society and the Millennium Cohort Study), to assess whether causality can be determined, and to what extent;
- Applying the methodology to a dataset that captures the impacts of COVID-19; and
- Using the methodology as part of a mixed methods study in a defined population group, combining the decomposition method to quantify and understand the health gap, and also using qualitative methods (such as in depth interviews) to further understand factors contributing to observed gaps

Application of the Decomposition Analysis across different countries, population groups, settings and health outcomes can develop the methodology further to help explain the health gap and its drivers better.

## Data availability statement

Publicly available datasets were analyzed in this study. This data can be found at: https://beta.ukdataservice.ac.uk/datacatalogue/series/series?id=2000035.

## Ethics statement

This is a secondary analysis of National Survey for Wales data which was accessed through the UK Data Service. Ethical review and approval was not required for the study on human participants in accordance with the local legislation and institutional requirements. The patients/participants provided their written informed consent to participate in this study.

## Author contributions

JA conceptualized and developed the manuscript. JA, AC-R, and OD performed the mapping of survey variables to WHO health equity framework. JA and OD performed data analysis and subsequent quality check. AC-R, MD, RM, and LM reviewed and contributed to the writing of the paper. Manuscript has been amended by JA following reviewer feedback. All authors contributed to the article and approved the submitted version.
