# Supplementary material for: Understanding health inequalities in Wales using the Blinder-Oaxaca decomposition method
Source: Front Public Health. 2022 Dec 15;10:1056885. doi: 10.3389/fpubh.2022.1056885 (PMC9797964; doi:10.3389/fpubh.2022.1056885)

**Supplementary material**

**Figure 1** Five essential conditions for healthy prosperous lives for all – WHO HESRi framework showing the different types of policies across sectors to address the wider determinants of health


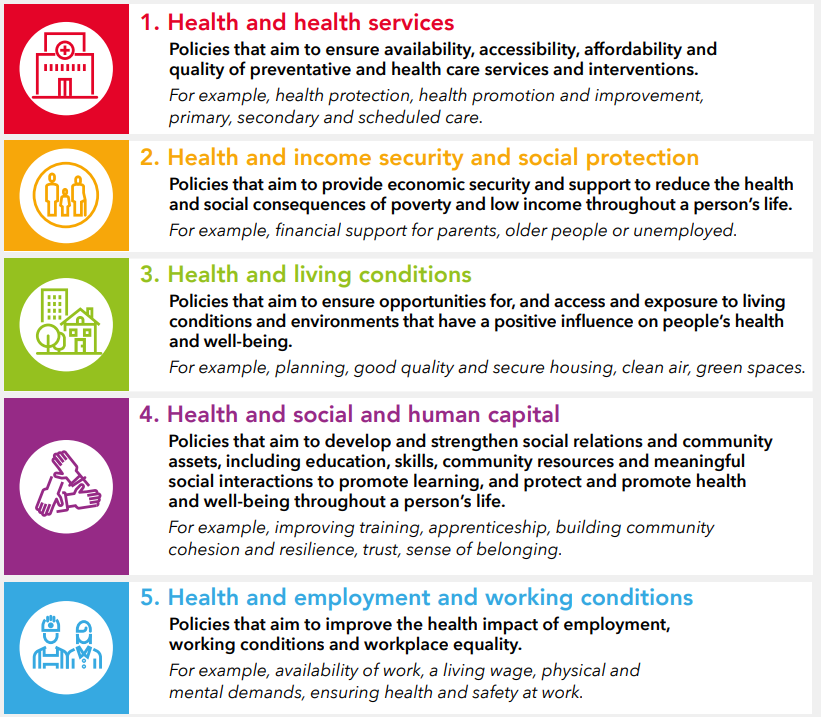


**Table 1** Mapping of survey question to analysis variables

| **Description** | **Response** | **Analysis value** |
| --- | --- | --- |
|  |  |  |
| **Outcomes** | | |
| Health in general | Very good | 0 |
|  | Good | 0 |
|  | Fair | 1 |
|  | Bad | 1 |
|  | Very bad | 1 |
| Well-being - Overall satisfaction with life (0-10 scale) | 0 | 1 |
|  | 1 | 1 |
|  | 2 | 1 |
|  | 3 | 1 |
|  | 4 | 0 |
|  | 5 | 0 |
|  | 6 | 0 |
|  | 7 | 0 |
|  | 8 | 0 |
|  | 9 | 0 |
|  | 10 | 0 |
| Warwick-Edinburgh Mental Well-being Scale - Grouped | Low well-being (14-44) | 1 |
|  | Medium well-being (45-57) | 0 |
|  | High well-being (58-70) | 0 |
| **Stratification factors** | | |
| Has a limiting long-standing illness, disability or infirmity | Yes | 1 |
|  | No | 0 |
| Do you make regular savings of £10 a month or more | We / I have this | 0 |
|  | We / I would like to have this but cannot afford this at the moment | 1 |
|  | We / I do not want / need this at the moment | 0 |
| Material deprivation | In material deprivation | 1 |
|  | Not in material deprivation | 0 |
| **Employment and working conditions** | | |
| Hours usually worked in main job (paid and unpaid) | 15 or less | 0 |
|  | 16 - 30 | 0 |
|  | 31 - 48 | 0 |
|  | 49 or more | 1 |
| Overall satisfaction with present job | Low (0-4) | 1 |
|  | Medium (5-6) | 0 |
|  | High (7-8) | 0 |
|  | Very high (9-10) | 0 |
| **Health services** | | |
| Overall satisfaction with the state of health services in Wales (0 -10 scale) | 0 | 0 |
|  | 1 | 0 |
|  | 2 | 0 |
|  | 3 | 0 |
|  | 4 | 1 |
|  | 5 | 1 |
|  | 6 | 1 |
|  | 7 | 1 |
|  | 8 | 1 |
|  | 9 | 1 |
|  | 10 | 1 |
| **Income security and social protection** | | |
| Keeping up with bills | Keeping up with all bills and commitments without any difficulties | 0 |
|  | Keeping up with all bills and commitments but it is a struggle from time to time | 0 |
|  | Keeping up with all bills and commitments but it is a constant struggle | 1 |
|  | Falling behind with some bills or credit commitments | 1 |
|  | Having real financial problems and have fallen behind with many bills or credit commitments | 1 |
|  | Have no bills | 0 |
| Respondent currently in paid work (either full-time or part-time) | Yes | 0 |
|  | No | 1 |
| Has household received food from a food bank in the last 12 months / Has household received food from a food bank in the last 12 months? (CASI) | Yes | 1 |
|  | No | 0 |
| **Living conditions** | | |
| Household type | Single pensioner (no children) | 1 |
|  | Married couple pensioner (no children) | 0 |
|  | Single person, not a pensioner (no children) | 1 |
|  | Two adult household with children | 0 |
|  | Two adult household (up to one pensioner) without children | 0 |
|  | Single parent household | 0 |
|  | Other households | 0 |
| Household has access to the internet | Yes | 0 |
|  | No | 1 |
| Overall satisfaction with local area as a place to live | Very satisfied | 0 |
|  | Fairly satisfied | 0 |
|  | Neither satisfied nor dissatisfied | 0 |
|  | Fairly dissatisfied | 1 |
|  | Very dissatisfied | 1 |
| People feeling safe (at home, walking in the local area, and travelling) | Feel safe | 0 |
|  | Don't feel safe | 1 |
| **Social and human capital** | | |
| Participating in any activity | Yes | 1 |
|  | No | 0 |
| Participation in sporting activities three or more times a week | Yes | 1 |
|  | No | 0 |
| There are many people I can trust completely | Yes | 1 |
|  | More or less | 1 |
|  | No | 0 |
| People who volunteer (formally or informally) | Yes | 1 |
|  | No | 0 |
| Have a sense of community (belonging; different backgrounds get on, treat with respect) | Yes | 1 |
|  | No | 0 |
| Highest level of qualification | Higher degree / postgraduate qualifications | 0 |
|  | First degree | 0 |
|  | Diplomas, etc. | 0 |
|  | A/AS level | 0 |
|  | Trade apprenticeships | 0 |
|  | O level / GCSE grades A-C, etc. | 0 |
|  | O level / GCSE D-G | 0 |
|  | Foreign qualifications | 0 |
|  | Other qualifications | 0 |
|  | No qualifications | 1 |

**Table 2** Blinder-Oaxaca decomposition of low life satisfaction, stratified by “Do you make regular savings of £10 a month or more”, non-pensioner adults (aged 16-65), Wales, 2016-17 to 2019-20


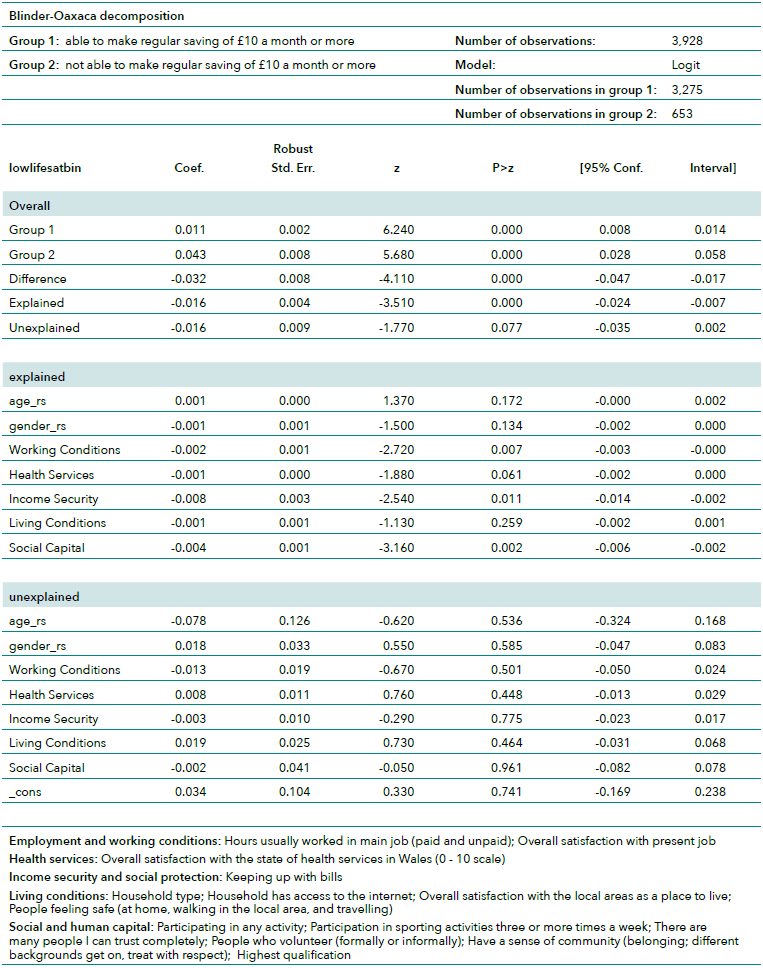


**Table 3** Blinder-Oaxaca decomposition of low mental well-being, stratified by “Do you make regular savings of £10 a month or more”, non-pensioner adults (aged 16-65), Wales, 2016-17 to 2019-20


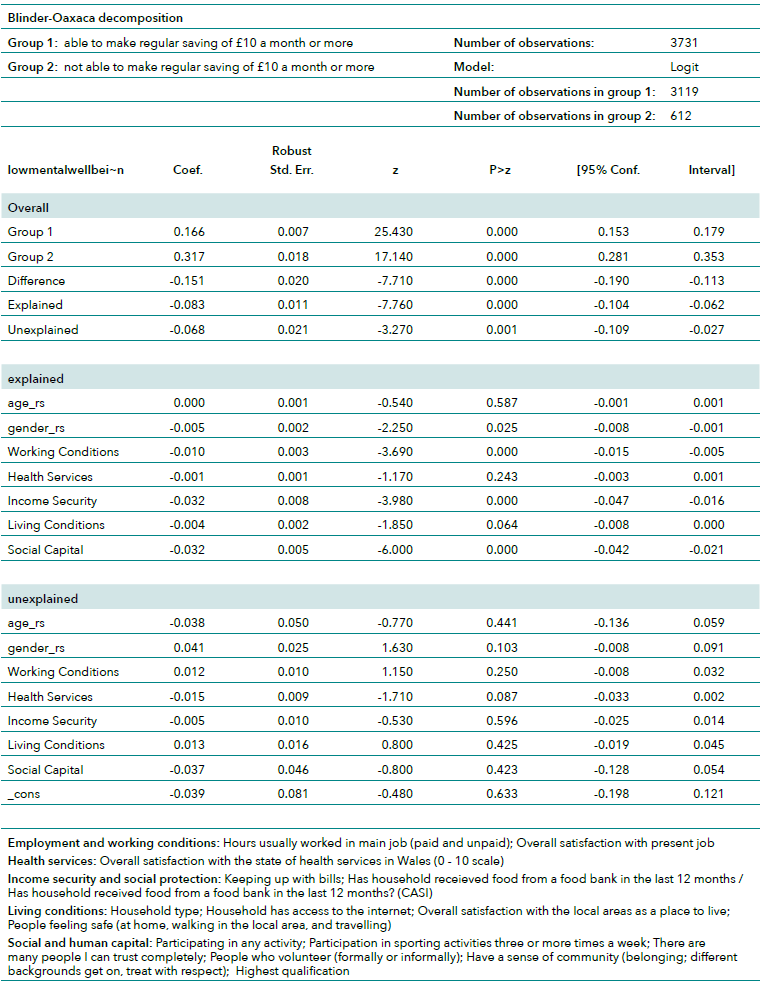


**Table 4** Blinder-Oaxaca decomposition of fair/poor health, stratified by “Do you make regular savings of £10 a month or more”, non-pensioner adults (aged 16-65), Wales, 2016-17 to 2019-20


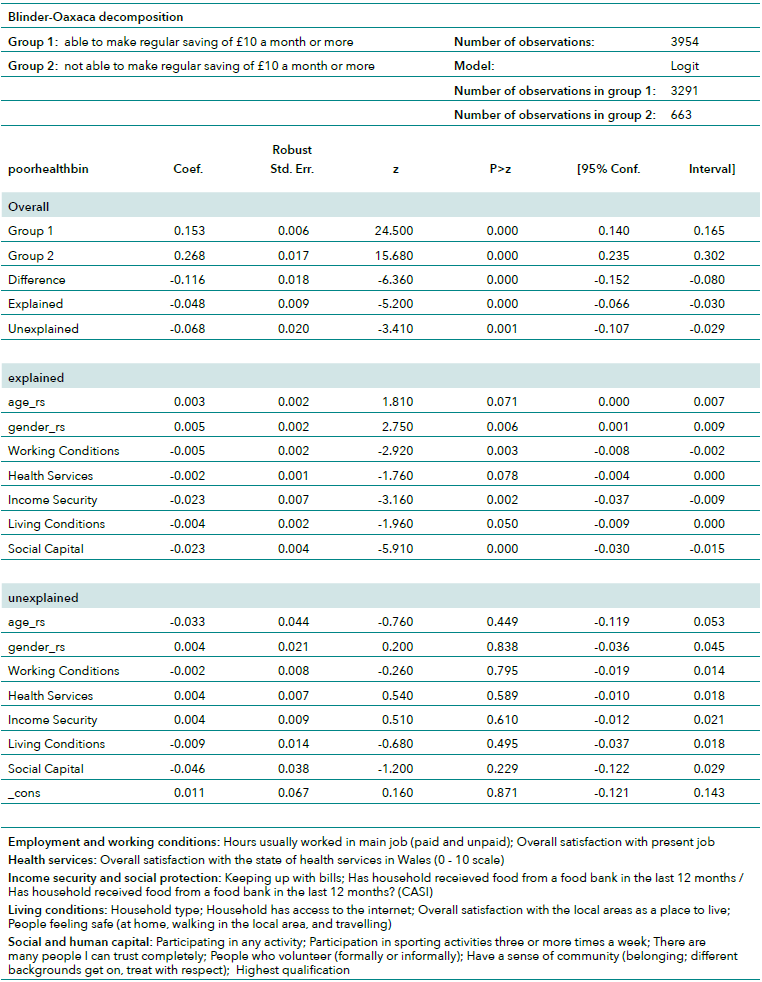


**Table 5** Blinder-Oaxaca decomposition of low life satisfaction, stratified by material deprivation, persons aged 16+, Wales, 2016-17 to 2019-20


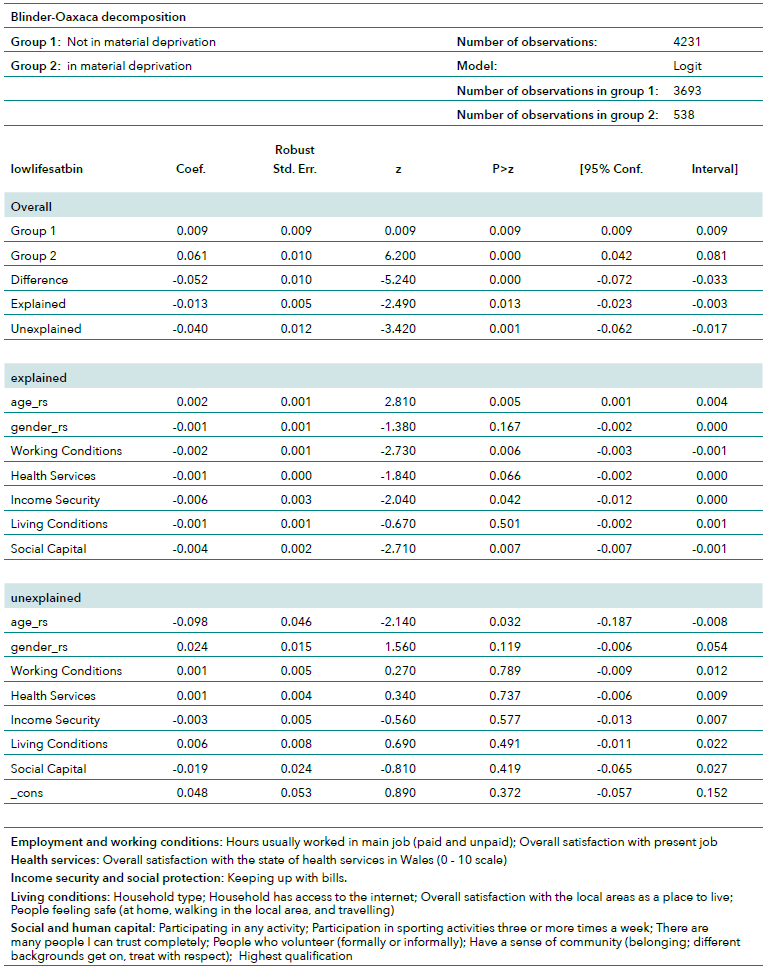


**Table 6** Blinder-Oaxaca decomposition of low mental well-being, stratified by material deprivation, persons aged 16+, Wales, 2016-17 to 2019-20


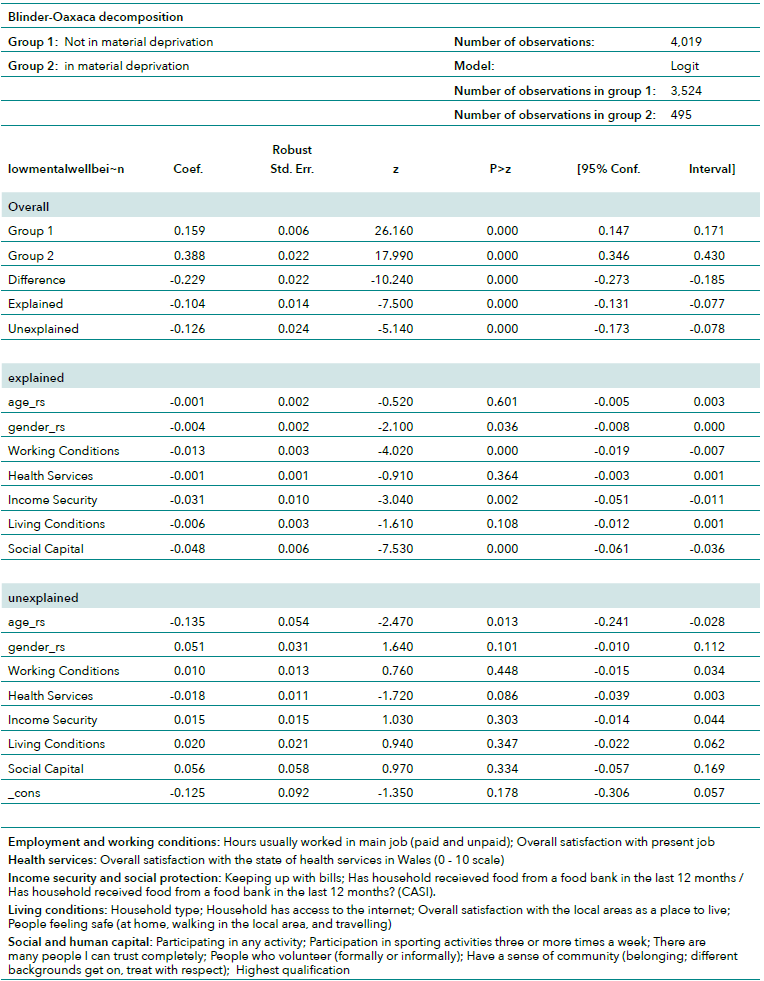


**Table 7** Blinder-Oaxaca decomposition of low fair/poor health, stratified by material deprivation, persons aged 16+, Wales, 2016-17 to 2019-20


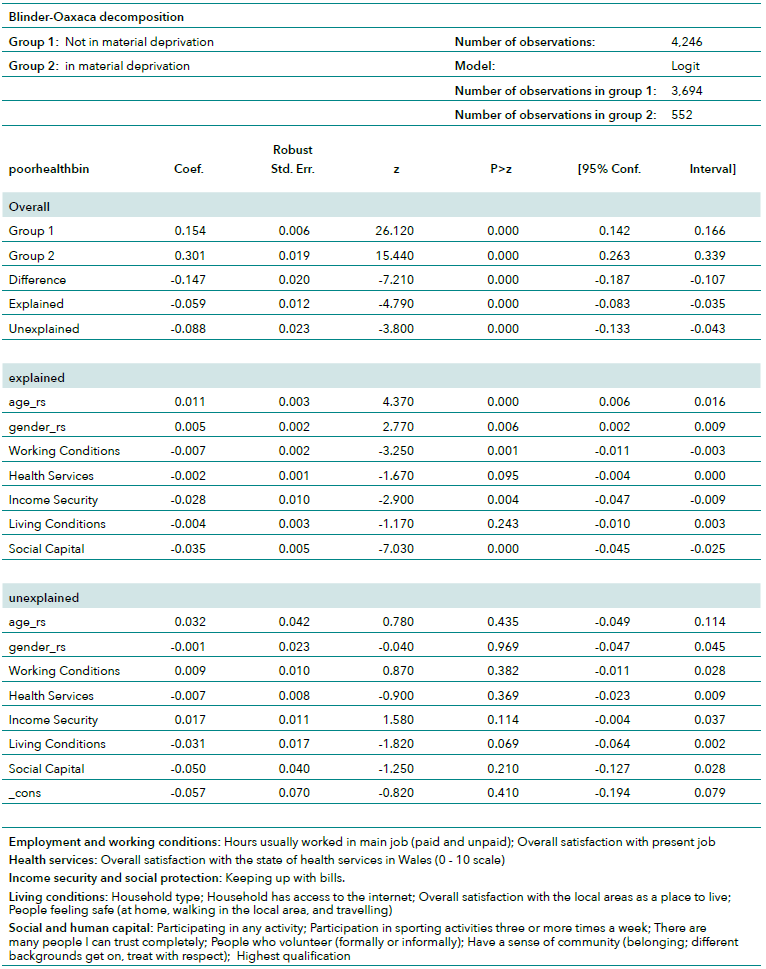


**Table 8** Blinder-Oaxaca decomposition of low life satisfaction, stratified by limiting long-standing illness, disability or infirmity, persons aged 16+, Wales, 2016-17 to 2019-20


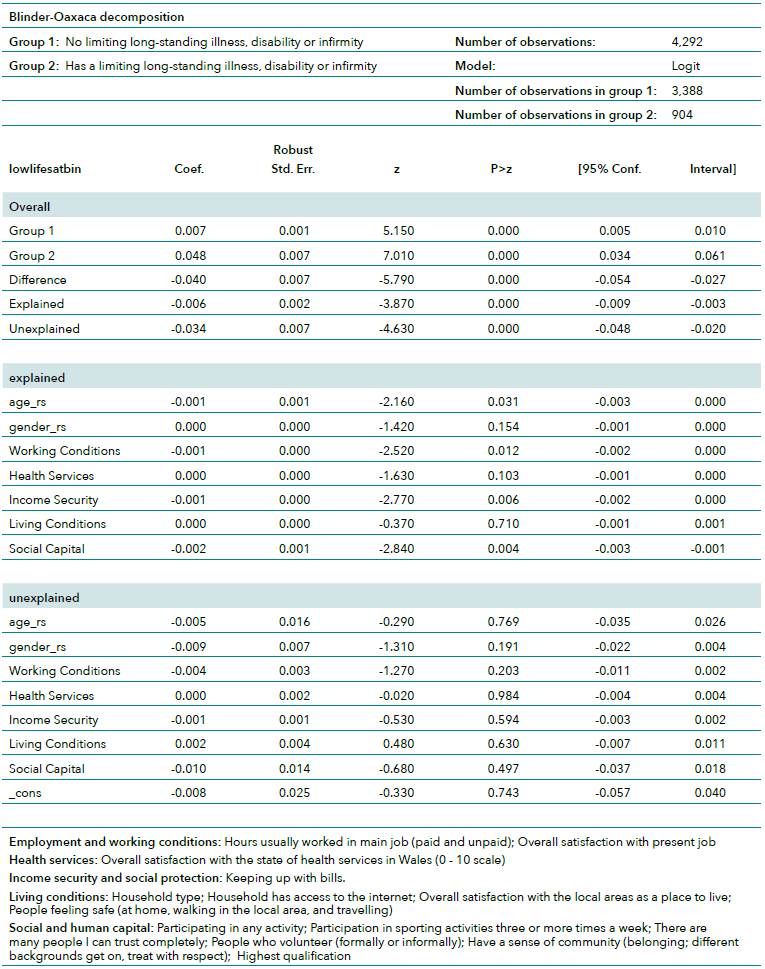


**Table 9** Blinder-Oaxaca decomposition of low mental well-being, stratified by limiting long-standing illness, disability or infirmity, persons aged 16+, Wales, 2016-17 to 2019-20


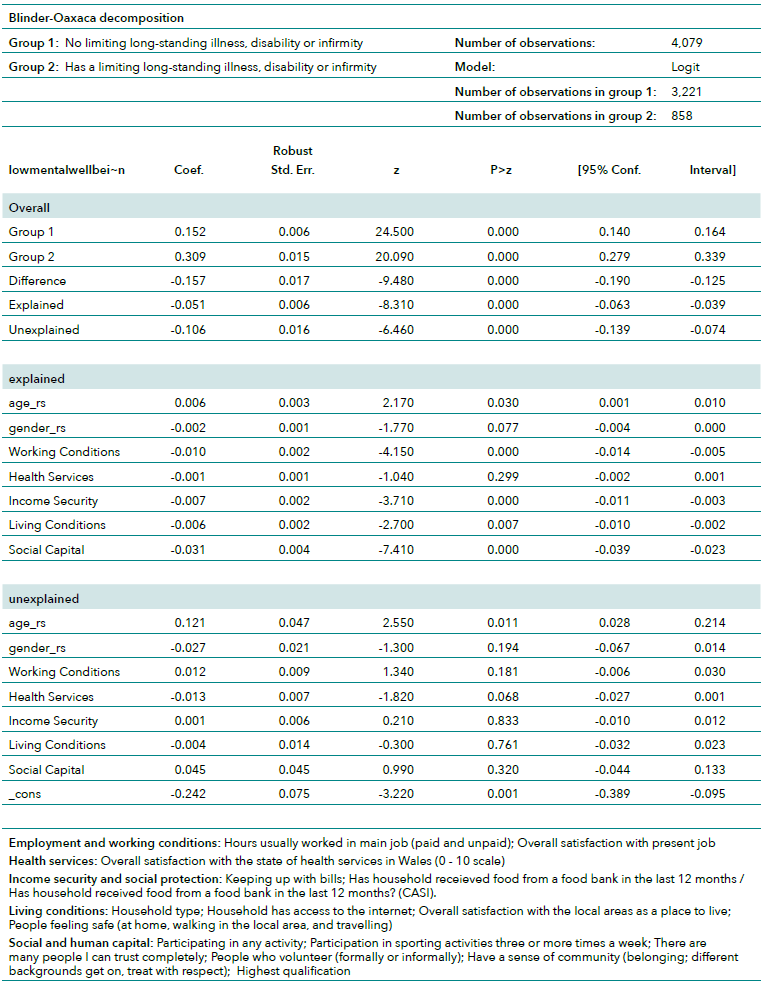

Supplement: Supplementary file 1 [file Data_Sheet_1.docx]
